# Comparison of S-ketamine and midazolam for intravenous preoperative sedative and anxiolytic effects in preschool children: study protocol for a randomized controlled clinical trial

**DOI:** 10.1186/s13063-023-07767-2

**Published:** 2023-11-13

**Authors:** Meng-Qiu Zhang, Ming-Zhe Xu, Yi He, Yong-Wei Su, Jun Ma, Yun-Xia Zuo

**Affiliations:** Department of Anaesthesiology, West China Hospital, Sichuan University, Chengdu, 610041 China

**Keywords:** S-ketamine, Midazolam, Preschool children, Randomized controlled trial

## Abstract

**Background:**

Preoperative anxiety management is gaining particular attention in paediatric anaesthesia. Pharmacological and non-pharmacological resorts can be implemented to address this special issue. Despite the various approaches currently used for preoperative sedation in children, the different sedative and anti-anxiety effects between the newly marketed anaesthetic, S-ketamine, and the traditional sedative, midazolam, are still unclear.

**Methods:**

This is a patient- and assessor-blinded randomized controlled clinical trial. Participants (*n* = 110) will receive S-ketamine (0.5 mg/kg) or midazolam (0.08 mg/kg) intravenously administrated at a ratio of 1:1 in the anaesthesia holding area. The primary outcome of this study is the sedative effect evaluated via the change in the modified Yale preoperative anxiety scale. It will be performed at two timepoints: in the pre-anaesthetic holding area before premedication (baseline, marked as T0) and about 5 min after premedication in the operating room without the existence of their guardians (marked as T1). Our secondary objectives include the parent separation anxiety score, postoperative agitation, caregivers’ and anaesthesia care providers’ satisfaction, and mask compliance.

**Discussion:**

This randomized controlled trial is the first study to compare the anti-anxiety effect of intravenous S-ketamine and midazolam. We will provide a new approach for the clinical management of preoperative anxiety in preschool children posted for elective surgery.

**Trial registration:**

ChiCTR2300069998. Registered on 30 March 2023.

**Supplementary Information:**

The online version contains supplementary material available at 10.1186/s13063-023-07767-2.

## Background

Children often present with different forms of anxiety, such as nervousness, crying, and non-cooperation prior to anaesthesia and surgery, preschool children in particular (aged 2 to 6 years). According to a cross-sectional survey study conducted in China, the incidence of preoperative anxiety in preschool children assessed by using the Modified Yale Preoperative Anxiety Scale (mYPAS) was 67.6% [1], which was comparable to that observed in other countries [2]. This could result in prolonged anaesthesia induction time, increasing aspiration and postoperative agitation risks, and even increasing the consumption of analgesics, which may, in turn, be associated with postoperative maladaptive behaviour habits, such as new onset enuresis, feeding difficulties, apathy and withdrawal, and sleep disturbances [3, 4]. Furthermore, all those consequences would negatively affect children’s recovery and physical and mental health [5, 6]. With the development of the concept of enhanced recovery after surgery, appropriate preoperative sedation is a necessary and invaluable tool for paediatric anaesthetists [7]. Therefore, drugs and non-drug intervention strategies have been proposed to prevent emotional and psychological injuries and minimize the potentially traumatic impact of anaesthesia and surgery in children with preoperative anxiety. Although there are many non-drug intervention measures, such as playing music or using tablets to play suitable cartoons to divert children’s attention, virtual reality, carrying children’s favourite toys, parental presence during the induction of anaesthesia, and medical personnel dressing up as clowns [8–10], sedative premedication is one of the most commonly used interventional techniques for the prevention and treatment of childhood preoperative anxiety [11]. Various agents have been advocated as premedication to allay anxiety and facilitate the smooth separation of children from their parents. Thus, an optimal agent for premedication in young children is crucial. Midazolam, which has the characteristics of high efficiency, high water solubility and anterograde amnesia, is a short-acting benzodiazepine central nervous system depressant with rapid onset. It is widely used and can effectively alleviate children’s preoperative anxiety, tension, and fear [12]. However, the onset time of midazolam is different among individuals. Its adverse reactions included nausea and vomiting, panic with disorientation, paradoxical reaction, and agitation during anaesthesia emergence [13–15]. While premedication in children is the first choice that for minimizing psychological trauma related to anaesthesia and surgery, the way of administration is mainly intranasally or orally because of the poor acceptance of children of an intravenous line before anaesthesia [16, 17]. In the literature thus far, only a few randomized clinical studies have assessed the efficacy of intravenous midazolam for preoperative sedation in paediatric individuals undergoing elective surgery [18, 19].

S-ketamine (esketamine), which is the S ( +) enantiomer of ketamine, is a new anaesthetic for Chinese anaesthetists and has becoming a popular and important ingredient for multimodal analgesia regimes because of its strong analgesic effects and the advantage of retaining spontaneous breathing. Compared with traditional racemic ketamine, S-ketamine is twice as strong as racemic ketamine in potency and about three times more potent than R-ketamine [20, 21]. Until now, the different sedative and anti-anxiety effects between this newly marketed anaesthetic, S-ketamine, and the traditional sedative, midazolam, have not been clear. Thus, in order to relieve the anxiety emotion of preschool children preoperatively and improve the satisfaction of caregivers and medical staff, so as to provide a new resort for the optimization of preschool children’s perioperative comfort medical service, we conducted this trial to compare the preoperative sedative and anxiolytic effects between intravenous S-ketamine and midazolam in preschool children.

## Method

### Study design and setting

**Fig. 1:**
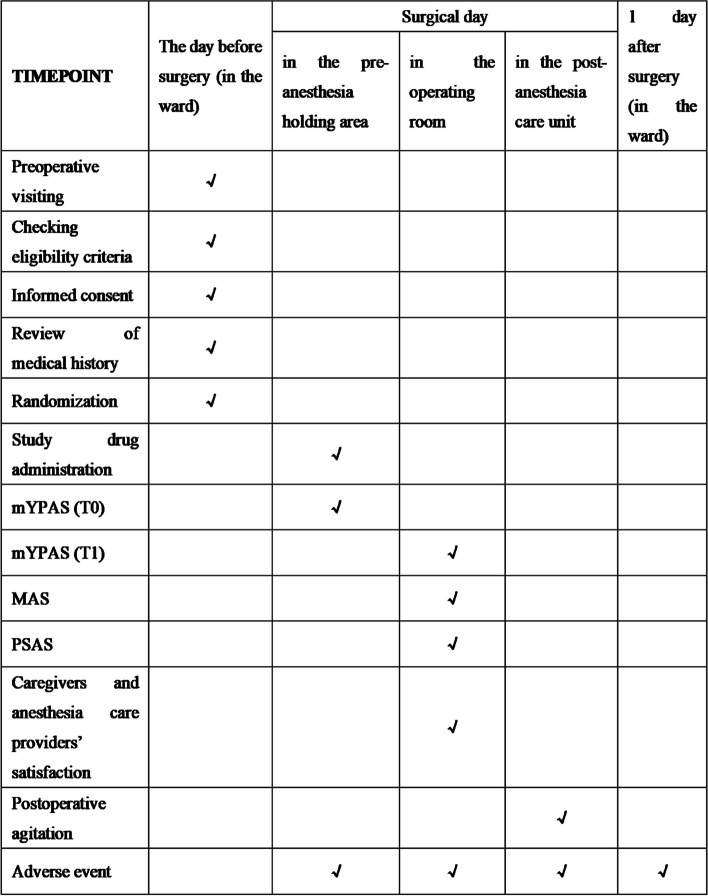
The schedule of major events that follows the Standard Protocol Items: Recommendation for Intervention Trials (SPIRIT) figure of enrolment, intervention, and assessment

This is a single-centre, double-blind, randomized, controlled clinical trial. A total of 110 participants from West China Hospital, Sichuan University will be recruited. This study protocol was approved by the Biomedical Research Ethics Committee of West China Hospital of Sichuan University on 15 March 2023 (approval number: 2023146) and registered in the Chinese Clinical Trials Registry on 30 March 2023 (registration number: ChiCTR2300069998). This clinical trial will be conducted following the ethical committee guidelines. Ethical committee’s approval will be required if there is a change of the protocol. The trial protocol is being conducted according to the recommendations of the Standard Protocol Items: Recommendations for Interventional Trials (SPIRIT) (Additional file 1: SPIRIT checklist). The goal of this study is to compare the sedative and anti-anxiety effects of two anaesthetics in preschool children, S-ketamine and midazolam, when administered intravenously before anaesthesia induction. The schedule of the major study events is shown in Fig. 1.

### Participants and eligibility criteria

Preschool children undergoing elective surgery will be screened, visited, and recruited in their ward the day before surgery. Their intravenous access has been established by an experienced nurse in a special room enriched with cartoon pictures and toys inside the ward before surgery. Topical anaesthesia and other non-pharm interventions are implemented to attenuate fear of needle puncture such as playing music, carrying children’s favourite toy, parental accompaniment, comfort, and encouragement. Participants meeting the following inclusion criteria and not meeting the exclusion criteria will be considered for enrolment before randomization.

Inclusion criteria:

1. Age between 2 and 6 years
2. American Society of Anesthesiologists (ASA) class graded at I–II
3. Elective surgery under general anaesthesia
4. An intravenous cannula was inserted preoperatively
5. Participants’ guardians or caregivers voluntarily participated and provided informed consent

Exclusion criteria:

1. Allergy to benzodiazepines and/or S-ketamine/ketamine
2. Difficult airway
3. Hepatic and renal insufficiency
4. Congenital heart abnormalities
5. Respiratory issues
6. Neurological diseases
7. Long-term use of sedative and analgesic agents before surgery

### Enrolment and consent

**Fig. 2:**
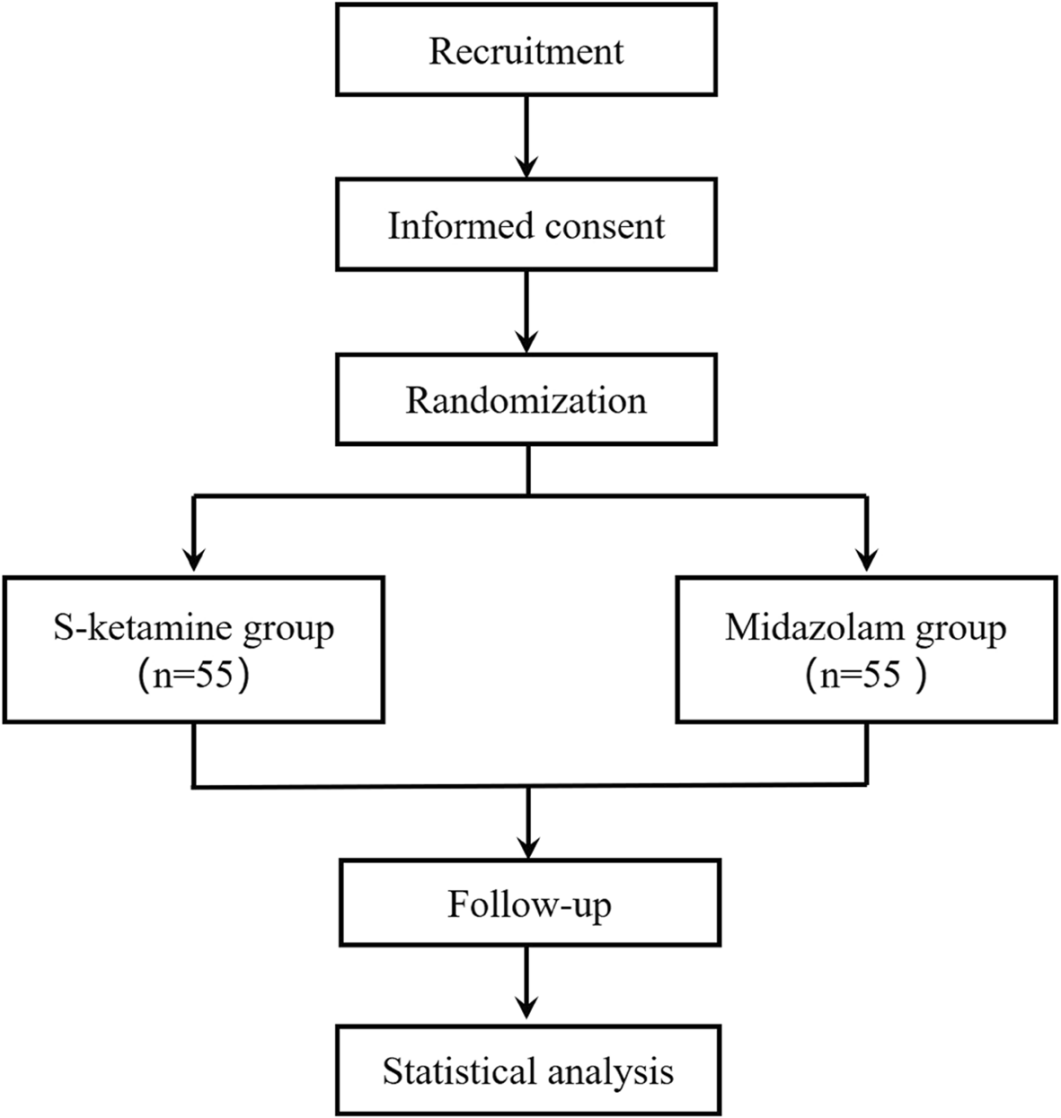
The CONSORT 2010 flow diagram

To enhance recruitment and adherence, electronic informed consent (e-IC) will be obtained after assessing the participants’ eligibility for inclusion. Compared to traditional paper IC, e-IC may improve participants’ comprehension and recall of information [22]. A short video clip containing necessary information about the trial will be shown to the legally acceptable representative (LAR). Subsequently, the LAR should sign and personally date the written e-IC form through a handwriting tablet face-to-face; it can be withdrawn at any time during the trial. Moreover, the LAR will be encouraged to contact the research team if they have any health concerns during the trial. Any serious adverse event, whether related to those two drugs under study or not, will be immediately reported and discussed. The flow chart of the study is shown in Fig. 2.

### Randomization and blinding

According to the random sequences generated by SPSS 26.0 (IBM, Chicago, IL, USA), the eligible participants will be randomly categorized into the S-ketamine group (group S) or the midazolam group (group M) in a 1:1 ratio per the randomized sequence table. Screening, obtaining assent, randomizing, and concealing will be conducted by a person not involved in the study the day before surgery. He will deliver the sealed, opaque, sequentially numbered envelopes to the anaesthesia care providers on the surgery day. The independent person in charge of data statistical analysis and the evaluators who monitored and recorded outcome measures will be blinded to the allocated intervention.

While the participants, indicator evaluator, data collectors, statistician, and surgeon will be unaware of the allocation until the study is completed, it is probably impossible to blind the anaesthesia caregivers due to the special clinical manifestation of S-ketamine, such as reduced blinking, rigidity, and expressionlessness. Moreover, for safety considerations, anaesthesia care providers are unblinded. In the case of medical emergencies requiring identification of the participants’ treatment, the investigators will be allowed to open the respective emergency envelope. As a consequence, the causation and compensation will be discussed. Whether it is related to the experimental drugs will also be identified.

### Interventions

Two drugs will be administered intravenously in the anaesthesia holding area: the atropine and S-ketamine or midazolam. For safety considerations, all the participants received an intravenous usage of 10 μg/kg atropine (H42021498, Huazhong Pharmaceutical Co., LTD.) for anticholinergic activity before administration of the experimental drugs. Then, S-ketamine (Jiangsu Hengrui Pharmaceutical Co., LTD.) 0.5 mg/kg or midazolam (Jiangsu Enhua Pharmaceutical Co., LTD.) 0.08 mg/kg will be intravenously administered 5 to 10 min before entering the operation room. The pulse rate and oxygen saturation will be monitored and recorded.

### Perioperative management

Anaesthesia care providers conducted routine anaesthetic care at their medical discretion regardless of the drugs used preoperatively. Standards for basic anaesthetic monitoring, which included blood pressure, electrocardiogram, oxygen saturation, heart rate, temperature, and end-tidal anaesthetic gas measurements, will be conducted to detect basic vital signs. During the surgery, blood pressure and heart rate are maintained within 20% of the baseline level. Mechanical ventilation respiratory frequency and tidal volume will be adjusted to control the pressure of end-tidal carbon dioxide within the range of 35 ± 5 mmHg. The relevant types of intraoperative nerve block are determined by the anaesthesia care provider. A patient-controlled analgesia pump will be used for intravenous self-analgesia according to the needs of the children. The children, intubated or not, will be transferred to the post-anaesthesia care unit, where they will be monitored and provided individualized medical care by an independent attending anaesthetist. Fentanyl is used as a rescue drug to mitigate the pain situation postoperatively. The children were discharged to the ward when complete recovery was achieved as Aldrete’s score reached 10 based on consciousness, mobility, breathing, blood pressure, and oxygen saturation.

### Data collection and outcomes

The assessment of outcomes will be performed by two independent trained anaesthetists. The collection of perioperative data will be conducted through the hospital information system. All data collectors will be specially trained and will be familiar with the assessment of the various scales. Periodically regular group meetings will be conducted to discuss the problem that we encountered during the progress. We shall also organize the trial data regularly to check for any missing data. The related data will be collected and compared on the paper case report form: demographic information, history of general anaesthesia, parental satisfaction, vital signs (heart rate, oxygen saturation), intubation time, and hospital stay. The enrolment rate (defined as the proportion of invited potential participants enrolled and/or the number of participants recruited) and feasibility of e-IC will be evaluated as well.

Our primary outcome is the change in preoperative paediatric anxiety from baseline as measured by the mYPAS. The mYPAS is a validated perioperative paediatric anxiety instrument to assess the anxiety with 5 items (activity, emotional expressivity, vocalizations, state of apparent arousal, and use of parents). This scale ranges from 23.3 to 100, with higher scores indicating greater anxiety [23]. The mYPAS is measured at two timepoints: in the pre-anaesthetic holding area before premedication (baseline, marked as T0) and about 5 min after premedication in the operating room without the existence of guardians (marked as T1).

The secondary outcomes include the following:

1. Parent separation anxiety scale (PSAS) [24]: anxiety score was determined when the child was separated from the parents according to four levels: (1) easy to separate, (2) sobbing but easy to cease, (3) crying loudly and difficult to stop but without holding the parents and not letting them go, and (4) crying loudly and holding the parents and not willing to let them go. Children who score 1 or 2 are considered to have “successful separation from parents”, and the number is recorded in both groups.
2. Postoperative agitation: the paediatric anaesthesia emergence delirium (PAED) scale will be implemented to measure the severity of children’s agitation in the recovery room [25]. A threshold score of 10 is considered a discriminator of the presence or absence of agitation and the need for treatment.
3. Caregivers’ and anaesthesia care providers’ satisfaction is assessed by a numeric rating scale (NRS: 0 ~ 10, an NRS score > 7 is considered “satisfactory”).
4. Mask compliance: a four-point mask acceptance score (MAS) [24] is used to assess the children’s acceptance of the mask: 1 point, very good (not afraid, cooperative, easy to accept the mask); 2 points, good (slight fear of mask, easy to comfort); 3 points, moderate (moderate fear of mask, difficult to calm through comfort); and 4 points, poor (afraid, crying or struggling). We consider “satisfactory” mask compliance when scored “1” or “2”. The number of children with “satisfactory” scores will also be recorded in both groups.
5. Drug-related adverse events: salivation, reflux, aspiration, respiratory depression (peripheral oxygen saturation < 90% during spontaneous breathing), laryngeal/bronchospasm, irritability or delirium, paradoxical reaction, etc.
6. Other outcomes: hemodynamic variables, anaesthesia and recovery time, hospital stay, and hospital costs.

### Sample size

According to a previously published study, the mean mYPAS score after premedication with midazolam is 37 ± 17 [26]. S-ketamine is expected to reduce it by 12 points, which is considered a score reduction clinically relevant [27]. According to alpha = 0.05 and power = 0.90, the sample size was *n* = 44 cases in each group with a ratio of 1:1 calculated by the PASS 15 software. Considering a shedding rate of 20%, a sample size of 55 cases in each group is finally needed.

### Statistical analysis

A full analysis set will be applied according to the principle of intention-to-treat analysis, which includes all the subjects who received the random allocation by using SPSS version 26.0. The outcomes will be analysed as randomized, regardless of protocol adherence. All statistical tests are conducted by two-sided test, and differences will be considered statistically significant if *P* ≤ 0.05. Data are displayed as *n* (%), mean (standard deviation), median (interquartile range), and differences in the median with confidence intervals. Kolmogorov–Smirnov and Levene’s tests will be used to analyse the normality and variance homogeneity of the outcome data. The continuous data are compared using the one-way analysis of variance or the non-parametric Kruskal–Wallis test. Categorical data are compared using chi-square tests (or Fisher’s exact probability test where numbers are small). If missingness is > 5% for any variable, the mean normal value of the patient group will be used for imputation. Formal interim analysis of primary and secondary outcomes is not planned because of the short-term duration and the low-risk intervention.

## Discussion

Despite the living environment, educational concepts, and the relationship between children and parents, the percentage of preoperative anxiety is similar and high in China [1] and other countries [2, 28]. This situation becomes more complicated by the psychological characteristics of 2 to 6-year-old preschool children. Children at this age are generally more variable, impulsive, and react unintentionally. They are aware of the existence of their guardians and more prone to fear and separation anxiety. Therefore, preoperative anxiety in preschool children demands more attention because children older than 7 years are more accepting and can be persuaded and tolerate separation.

There are extensive studies aimed at exploring the difference in preoperative sedation effects in children with midazolam, dexmedetomidine, and ketamine compared to other sedation methods [29–32]. In particular, several studies have compared the effects of midazolam and ketamine for oral premedication [33, 34]. They similarly concluded that the sedation degree was superior in the combination group (midazolam plus ketamine). In most studies, the route of premedication was oral or intranasal because of no placement of the indwelling needle before anaesthesia. Until now, only few studies have been conducted with intravenous form as premedication [35, 36]. When inpatient paediatric patients with a previously existing indwelling needle need to prepare for the operating room, intravenous premedication is superior in this situation. Sajedi and colleagues [36] recruited 90 paediatric patients aged from 6 months to 6 years and divided them into three groups to receive intravenous midazolam 0.1 mg/kg, or intravenous ketamine 1 mg/kg, or a combination of half doses of both. It was revealed that the combination group was superior to the other two groups. In addition to the administration of midazolam for preoperative sedation and anti-anxiety, S-ketamine is a new choice for paediatric anaesthetists. Marhofer et al. concluded that S-ketamine for rectal premedication (1.5 mg/kg) alone showed a poor anaesthetic effect and a frequent incidence of side effects during induction of anaesthesia via face mask compared with the combination of midazolam/S-ketamine and plain midazolam [37]. For autism spectrum disorder patients, oral premedication with S-ketamine plus midazolam also provided a satisfactory pre-anaesthetic sedation effect and facilitated intravenous line access [38]. In those previous studies, non-intravenous administration of S-ketamine was used as premedication under different circumstances. The sedative effect between intravenous S-ketamine and other sedatives in preschool children is not clear. Therefore, we designed this trial to compare the sedative effect of the newly approved anaesthetic, S-ketamine, with the traditional sedative agent, midazolam in preschool children. In this prospective randomized trial, anaesthesia care providers could not be blinded to the intervention group for obvious reasons. As a result, we cannot eliminate the risk of bias due to a lack of blinding. The strengths of the study include the randomized controlled design, pragmatic implementation, and utilization of established validated outcome scales that would reduce observer bias.

We predict that children who receive S-ketamine will experience less anxiety than those who receive midazolam during the preoperative period, and the results of our trial could provide further insights in the management of preoperative sedation.

## Trial status

The trial was started after we obtained the approval of the local ethics committee and registered in the Chinese Clinical Trials Registry. The approximate date when recruitment will be completed is approximately December 2023.

### Supplementary Information

**Additional file 1. **SPIRIT Checklist for Trials.

## Data Availability

All the paper case report forms will be collected and reserved by a special data manager of our department. Personal information about participants will be kept strictly confidential. The statistical database will be available upon reasonable request after the publication of the study results.

## Abbreviations

ASA: American Society of Anesthesiologists
e-IC: Electronic informed consent
LAR: Legally acceptable representative
mYPAS: Modified Yale preoperative anxiety scale
PSAS: Parent separation anxiety scale
PAED: Paediatric anaesthesia emergence delirium
MAS: Mask acceptance score
