# Supplementary material for: Comparison of S-ketamine and midazolam for intravenous preoperative sedative and anxiolytic effects in preschool children: study protocol for a randomized controlled clinical trial
Source: Trials. 2023 Nov 13;24:724. doi: 10.1186/s13063-023-07767-2 (PMC10644481; doi:10.1186/s13063-023-07767-2)
Supplement: Supplementary file 1 — Additional file 1. SPIRIT Checklist for Trials. [file 13063_2023_7767_MOESM1_ESM.pdf]

SPIRIT-Outcomes 2022 Extension items only (for separate completion of SPIRIT 2013 and SPIRIT-Outcomes 2022 items)[a]

Section	Item No.	SPIRIT-Outcomes 2022 item	Location Reported[b]
**Methods: Participants, interventions, and outcomes**			
**Outcomes**	12.1	Provide a rationale for the selection of the domain for the trial's primary outcome	Line 14-19, Page 4.
	12.2	If the analysis metric for the primary outcome represents within-participant change, define and justify the minimal important change in individuals	not applicable
	12.3	If the outcome data collected are continuous but will be analyzed as categorical (method of aggregation), specify the cutoff values to be used	Line 20-22, Page 9; Line 25-28, Page 9; Line 3-4， Page 10
	12.4	If outcome assessments will be performed at several time points after randomization, state the time points that will be used for analysis	LIne 12-14, Page 9
	12.5	If a composite outcome is used, define all individual components of the composite outcome	not applicable
**Sample size**	14.1	Define and justify the target difference between treatment groups (eg, the minimal important difference)	Line 13-17, Page 10
**Methods: Data collection, management, and analysis**			
**Data collection methods**	18a.1	Describe what is known about the responsiveness of the study instruments in a population similar to the study sample	not applicable
	18a.2	Describe who will assess the outcome (eg, nurse, parent)	Line 26-27, Page 8
**Statistical methods**	20a.1	Describe any planned methods to account for multiplicity in the analysis or interpretation of the primary and secondary outcomes (eg, coprimary outcomes, same outcome assessed at multiple time points, or subgroup analyses of an outcome)	Line 19, Page 10

[a]It is strongly recommended that this checklist be read in conjunction with the SPIRIT (Standard Protocol Items: Recommendations for Interventional Trials) Statement paper for important clarification on the items. Amendments to the protocol should be tracked and dated. The SPIRIT checklist is copyrighted by the SPIRIT Group under the Creative Commons "Attribution-NonCommercial-NoDerivs 3.0 Unported" license and is reproduced with permission.
[b]Indicates page numbers and/or manuscript location: to be completed by authors during trial protocol development.

Please cite as: Butcher NJ, Monsour A, Mew EJ, et al. Guidelines for reporting outcomes in trial protocols: the SPIRIT-Outcomes 2022 extension. JAMA. Published online December 13, 2022. doi:10.1001/jama.2022.21243
